# Comparative Study of Long-Term Outcomes of Laparoscopic Liver Resection versus Radiofrequency Ablation for Single Small Hepatocellular Carcinoma Located in Left Lateral Segments of the Liver

**DOI:** 10.3390/medicina59061063

**Published:** 2023-06-01

**Authors:** MeeYoung Kang, Jai Young Cho, Ho-Seong Han, Yoo-Seok Yoon, Hae Won Lee, Boram Lee, Yeshong Park, Jinju Kim, Chang Jin Yoon

**Affiliations:** 1 Department of Surgery, Seoul National University Bundang Hospital, Seoul National University College of Medicine, Seoul 13620, Republic of Korea; 2 Department of Radiology, Seoul National University Bundang Hospital, Seoul National University College of Medicine, Seoul 13620, Republic of Korea

**Keywords:** hepatocellular carcinoma, laparoscopic resection, radiofrequency ablation

## Abstract

*Background and Objectives*: Laparoscopic liver resection (LLR) is now widely recognized as the primary surgical option for hepatocellular carcinomas (HCC) smaller than 3 cm located in the left lateral segment of the liver. Nevertheless, there is a scarcity of studies comparing laparoscopic liver resection with radiofrequency ablation (RFA) in these cases. *Materials and Methods*: We retrospectively compared the short- and long-term outcomes of Child–Pugh class A patients who underwent LLR (*n* = 36) or RFA (*n* = 40) for a newly diagnosed single small (≤3 cm) HCC located in the left lateral segment of the liver. *Results:* Overall survival (OS) was not significantly different between the LLR and RFA groups (94.4% vs. 80.0%, *p* = 0.075). However, disease-free survival (DFS) was better in the LLR group than in the RFA group (*p* < 0.001), with 1-, 3-, and 5-year DFS rates of 100%, 84.5%, and 74.4%, respectively, in the LLR group vs. 86.9%, 40.2%, and 33.4%, respectively, in the RFA group. The hospital stay was significantly shorter in the RFA group than in the LLR (2.4 vs. 4.9 days, *p* < 0.001). The overall complication rate was higher in the RFA group than in the LLR group (15% vs. 5.6%). In patients with an α-fetoprotein level of ≥20 ng/mL, the 5-year OS (93.8% vs. 50.0%, *p* = 0.031) and DFS (68.8% vs. 20.0%, *p* = 0.002) rates were greater in the LLR group. *Conclusions:* LLR showed superior OS and DFS compared to RFA in patients with a single small HCC situated in the left lateral segment of the liver. LLR can be considered for patients with an α-fetoprotein level of ≥20 ng/mL.

## 1. Introduction

Hepatocellular carcinoma (HCC) ranks seventh among the most prevalent types of cancer globally and is the second most common cause of cancer-related mortality [1]. With the continued development of early screening and treatment technologies, HCC has evolved over the past decade from an incurable type of cancer into a treatable, controllable disease [2]. Serum α-fetoprotein (AFP) is the most widely used serological marker to establish a diagnosis of HCC and is included in international guidelines for HCC surveillance [3]. An elevated serum AFP level is usually associated with a high risk of developing HCC and a poor prognosis [4].

Early detection of HCC allows radical treatment by surgical resection, liver transplantation, or percutaneous ablation. Currently, radiofrequency ablation (RFA) competes with liver resection and liver transplantation as the primary treatment modality for small HCCs. As stated in the BCLC classification, in the very early stage, HCC (single nodules of ≤2 cm) ablation is a gold standard for Child-Pugh A, ECOG 0 patients. Following the Milan criteria (single HCC of ≤5 cm or up to 3 nodules of <3 cm), the best treatment for HCC is liver transplantation, but this procedure is limited due to the difficulty of finding suitable donors [5]. The European Association for the Study of the Liver (EASL) [3] and the American Association for the Study of Liver Disease (AASLD) [6] clinical practice guidelines recommend surgical resection and percutaneous ablation for patients with early and moderate HCC. The Korean Liver Cancer Association (KLCA) and National Cancer Center (NCC) of Korea guidelines for HCC indicate that RFA has an equivalent survival rate, a higher local tumor progression rate, and a lower complication rate compared with hepatic resection in patients with a single nodular HCC ≤3 cm in diameter [7].

LLR has become a popular treatment choice for HCC in the left lateral segment due to the progress in surgical techniques, resulting in lower morbidity, shorter hospital stays, and better patient outcomes than conventional open resection [8].

Several studies have compared the outcomes of laparoscopic liver resection (LLR) and radiofrequency ablation (RFA) for the treatment of hepatocellular carcinoma (HCC), demonstrating favorable short- and long-term results for both procedures. However, there remains a knowledge gap regarding the optimal treatment approach for non-cirrhotic patients with small early-stage HCC (Child-Pugh A). Thus, the aims of this study were to retrospectively evaluate the long-term outcomes of laparoscopic left lateral resection (LLR) compared with percutaneous RFA in patients with Child–Pugh class A liver cirrhosis for the treatment of a single small HCC located in the left lateral segment. We also compared the outcomes between these procedures in patients with an AFP level of ≥20 ng/mL.

## 2. Materials and Methods

This study was approved by the Ethics Committee of Seoul National University Bundang Hospital (approval number: B-2304-821-101, 2023-03-21) and conducted in accordance with the Declaration of Helsinki.

### 2.1. Inclusion Criteria

We reviewed the medical records of patients with newly diagnosed HCC who were admitted to Seoul National University Bundang Hospital (Seoul, South Korea) between 2009 and 2018. Patients who met the following criteria were included in this study: (a) Child–Pugh class A liver cirrhosis or non-cirrhotic liver; (b) single tumor ≤3 cm in diameter without metastasis or vascular invasion; (c) new diagnosis without a history of surgical resection or non-surgical treatment for HCC; and (d) HCC located in the left lateral segment (S2, S3). Patients aged <18 years, with a tumor >3 cm in diameter, a tumor located in a segment other than the left lateral segment (S2 or S3), other pathological or radiological malignancy of the liver, and those lost to follow-up after hepatectomy or RFA were excluded from this study. Patients with advanced liver cirrhosis (Child–Pugh class B or C) and patients who underwent liver transplantation during the study period were also excluded.

During the study period, patients admitted to our department underwent pretreatment screening examinations, including blood tests (blood biochemistry and AFP), chest radiography, abdominal computed tomography (CT) and/or magnetic resonance imaging (MRI). HCC was diagnosed according to the EASL or AASLD clinical practice guidelines, based on the histology or typical features of HCC observed by four-phase multidetector CT or dynamic contrast-enhanced MRI.

Postoperative complications were scored according to the Clavien–Dindo classification. Postoperative or postprocedural morbidity and mortality were defined as events that occurred during the postoperative and postprocedural hospital stay or within 30 days after surgery or RFA.

### 2.2. RFA Procedure and Surgical Technique

RFA was performed using a flat-panel monoplane angiographic suite (Allura Xper, Philips Healthcare, Best, The Netherlands) under fluoroscopic guidance. A 17-gauge internally cooled electrode was used with an expandable 10-hook LeVeen needle (Boston Scientific Corporation, Marlborough, MA, USA). After an appropriate RF electrode entry site was marked on the patient’s skin under ultrasound guidance (Logiq E9, GE Healthcare, Republic of Korea), the RF electrode was advanced a few centimeters into the liver parenchyma aiming at the iodized oil that had accumulated in the index tumor under the fluoroscopic left lateral projection. The left lateral direction of the electrode was adjusted based on the lateral and oblique projections. The electrode direction was repeatedly adjusted by obtaining multiple fluoroscopic projections to ensure the RF electrode reached the tumor. At the end of the procedure, the electrode tract was cauterized to prevent bleeding and tumor seeding.

Surgical resection was carried out with the patient under general anesthesia and tilted to a 30° reverse Trendelenburg position with lithotomy. A 12-mm camera port was placed in the sub-umbilical position, after which pneumoperitoneum was established and maintained at <13 mmHg. Two 11-or 12-mm main working ports were inserted in the subcostal area where it meets the midclavicular line and epigastric area, respectively. A 5-mm port was then placed where the subcostal area meets the anterior axillary line. The scope technician stood in the middle, and the assistant was on the left side of the patient. A flexible scope was used, and intraoperative ultrasound of the liver was performed to determine the exact tumor location and to confirm the resection margin. Ultrasonic shears were used to transect the superficial hepatic parenchyma, and a laparoscopic Cavitron ultrasonic surgical aspirator (CUSA, Integra Lifesciences, Princeton, NJ, USA) was used to dissect the deeper parenchyma. When necessary, an Endo-GIA stapler (Medtronic, Tampa, FL, USA) was used to divide the liver parenchyma or glissonean pedicle.

### 2.3. Statistical Analyses

Statistical analyses were performed using SPSS software version 27.0 (SPSS). The two groups were compared using Student’s *t*-test for continuous data and the χ^2^ test or Fisher’s exact test for categorical data. All data are expressed as the mean ± standard deviation or as the median and range. Disease-free survival (DFS) and overall survival (OS) rates were calculated using the Kaplan–Meier method. Differences between the survival curves were assessed using the log-rank test. Variables that showed statistical significance in univariate analyses were included in multivariable analyses using Cox proportional hazard models. A value of *p* < 0.05 was considered statistically significant.

## 3. Results

### 3.1. Patient Characteristics

A total of 76 patients with an HCC ≤3 cm located in their left lateral segment (S2 and S3) were reviewed. Thirty-six patients initially underwent laparoscopic surgical resection of the left lateral segment (including left lateral resection, S2 or S3 segmentectomy or tumorectomy), and 40 were treated with percutaneous RFA. The median follow-up times for the LLR and RFA groups were 57 months (range 0–118 months) and 63 months (range 0–131 months). Table 1 provides the baseline characteristics of both groups. Most patients had hepatitis B virus-related HCC (83.3% in LLR vs. 77.5% in RFA); the proportions of patients with hepatitis virus- and alcohol-induced HCC were not significantly different between the LLR and RFA groups. Patients who underwent LLR had significantly higher AFP levels (*p* = 0.020) and larger tumors (*p* = 0.003) than patients who underwent RFA. The serum prothrombin time international normalized ratio (*p* = 0.003), albumin (*p* = 0.001), aspartate aminotransferase (*p* = 0.007), and alanine aminotransferase (*p* = 0.002) levels were significantly higher in the RFA group than in the LLR group.

### 3.2. Outcomes

**Figure 1:**
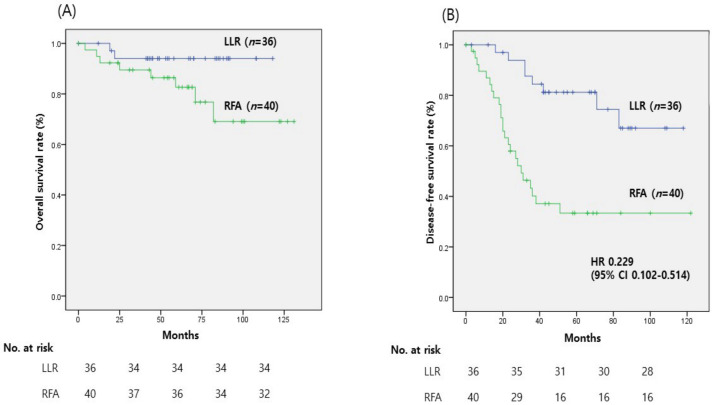
Survival curves of patients who underwent laparoscopic liver resection (LLR) or radiofrequency ablation (RFA) for the treatment of hepatocellular carcinoma. (**A**) Cumulative overall survival (OS) curves for patients. The OS rate was not significantly different between the two groups (*p* = 0.075). (**B**) Cumulative disease-free survival (DFS) curves for patients. DFS was significantly greater in the LLR group than in the RFA group (*p* < 0.001). The LLR group shows a HR of 0.229 (95% CI: 0.102–0.514) compared to RFA, indicating improved overall survival.

There was no significant difference in OS between the LLR and RFA groups (94.4% vs. 80.0%, *p* = 0.075). However, DFS was better in the LLR group than in the RFA group (*p* < 0.001), with 1-, 3-, and 5-year DFS rates of 100%, 84.5%, and 74.4%, respectively, in the LLR group vs. 86.9%, 40.2%, and 33.4%, respectively, in the RFA group (Figure 1). The proportion of patients who suffered early recurrence (within 6 months) was significantly higher in the RFA group than in the LLR group (10% vs. 0%, *p* = 0.044).

In the RFA group, 21 (52.5%) patients had intrahepatic recurrence, 1 (2.5%) patient had extrahepatic recurrence, and 1 (2.5%) had concurrent intrahepatic and extrahepatic recurrence. In the LLR group, eight (22.2%) patients had an intrahepatic recurrence, and one (2.8%) patient had concurrent intrahepatic and extrahepatic recurrence; none had extrahepatic recurrence only. The recurrence pattern significantly differed between the two groups (*p* = 0.023). Regarding hospital stay, patients who underwent LLR had a longer duration of hospitalization, with an average of 4.9 days, compared to 2.4 days in the RFA group (*p* < 0.001). Neither group had clinically relevant complications (Clavien–Dindo classification above IIIa). However, the overall complication, including CDC class I and II, was significantly high in the RFA group than in the LLR group (15% vs. 5.6%, *p* = 0.009) (Table 2).

### 3.3. Subgroup Analyses in Patients with an AFP Level of ≥20 ng/mL

The AFP level was ≥20 ng/mL in 16 of 36 patients in the LLR group and 10 of 40 patients in the RFA group (Table 3). Patients who underwent LLR had larger tumors (2 cm vs. 1.4 cm, *p* = 0.017) and higher AFP levels (443.4 vs. 106.3, *p* = 0.026) than patients who underwent RFA. The general liver function was better in the LLR group than in the RFA group. Neither group had clinically relevant complications (Clavien-Dindo classification above IIIa). However, the overall complication, including CDC class I and II, was significantly higher in the RFA group than in the LLR group. In the LLR group (*n* = 16), overall complications were observed in 2 patients (12.5%), while in the RFA group (*n* = 10), 1 patient (10%) experienced complications. The difference in overall complications was not statistically significant (*p* = 0.854). Organ injury occurred in one patient (6.25%) in the LLR group (liver), and in the RFA group, one patient (10%) experienced organ injury involving the stomach. No cases of fluid collection or other complications were reported in either group. Early recurrence, defined as recurrence within 6 months, was not observed in the LLR group, while one patient (10%) in the RFA group experienced early recurrence. The difference in early recurrence between the two groups was not statistically significant (*p* = 0.343). In terms of recurrence patterns, the LLR group had a higher proportion of no recurrence (68.8%) compared to the RFA group (20%). Intrahepatic recurrence was common in the RFA group (70%) compared to the LLR group (31.3%). One patient in the RFA group (10%) experienced extrahepatic recurrence. None of the patients in either group had both intrahepatic and extrahepatic recurrence. The difference in recurrence patterns between the two groups was statistically significant (*p* = 0.009). The hospital stay was significantly shorter for the RFA group than the LLR group (1.8 vs. 5.0 days, *p* < 0.001) (Table 4).

**Figure 2:**
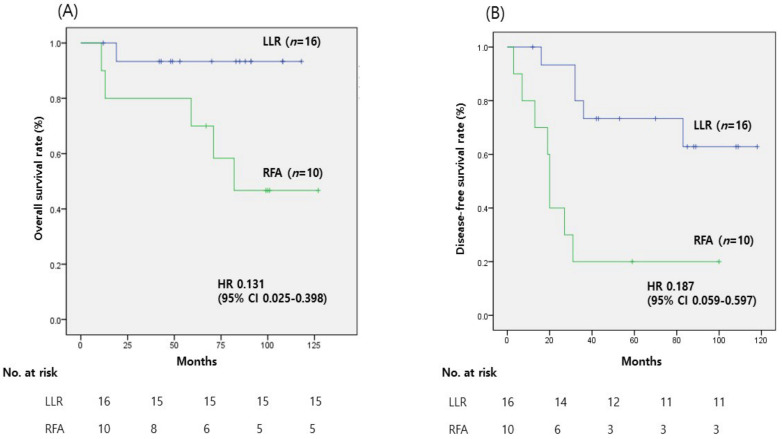
The survival curves of patients with α-fetoprotein (AFP) levels ≥20 ng/mL are shown. (**A**) In this subgroup, laparoscopic liver resection (LLR) demonstrated significantly improved overall survival (OS) compared to radiofrequency ablation (RFA) (*p* = 0.031). The LLR group shows a HR of 0.131 (95% CI: 0.025–0.398) compared to RFA, indicating improved overall survival. (**B**) Similarly, disease-free survival (DFS) was also higher in the LLR group than in the RFA group (*p* = 0.002). The LLR group shows a significantly lower recurrence rate compared to the RFA group, with an HR of 0.187 (95% CI: 0.059–0.597).

The 5-year OS (93.8% vs. 50.0%, *p* = 0.031) and DFS (68.8% vs. 20.0%, *p* = 0.002) rates were significantly greater in the LLR group than in the RFA group (Figure 2).

## 4. Discussion

In HCC located in the left lateral segment (S2, S3), LLR is less technically demanding and is associated with fewer postoperative complications and lower perioperative mortality than LLR of HCC located in other segments [9]. In non-cirrhotic patients with resectable HCC, liver resection is considered the gold-standard therapy.

A recent study reported a difference in survival rate after resection depending on the size of the HCC tumor and that early HCC with a small size (<5 cm) was associated with higher OS and DFS rates after resection compared with larger (≥5 to <10 cm) and huge (≥10 cm) HCC [10]. However, RFA has the advantage of being less invasive than surgical resection and had similar perioperative outcomes in patients with early small HCC ≤3 cm [11,12]. The most suitable treatment for non-cirrhotic (Child–Pugh A), small and early-stage HCC, is still controversial [13]. In addition, few studies have examined which treatment method is effective according to AFP in this patient group.

In this study of patients with Child–Pugh A liver cirrhosis who had a single small HCC located in the left lateral segment of the liver, LLR was associated with better OS and DFS compared with RFA.

In a recent study comparing the survival outcomes of liver resection (LR) and RFA in patients with CTP A and small HCC, LR was similar to RFA in terms of OS but showed better outcomes in terms of DFS [14]. We reviewed the findings of recent studies of a single small HCC located in the left lateral segment, in which LLR was associated with better survival and lower recurrence rates than RFA [15,16,17]. However, RFA was associated with shorter procedure time, fewer postoperative complications, and shorter hospital stays than LLR [13,18]. The type of treatment should be determined with regard to the severity of liver cirrhosis. In a previous study, liver resection offered advantages over RFA in terms of perioperative outcomes and survival in patients with Child–Pugh class A, but no differences in these outcomes were seen between liver resection and RFA in patients with Child–Pugh class B [19]. Therefore, further studies are needed to confirm whether RFA is a useful alternative to surgery for potentially resectable HCC.

One notable advantage of laparoscopic liver resection (LLR) is the ability to achieve complete tumor resection with negative margins, ensuring a lower risk of tumor recurrence compared to ablative techniques. Moreover, LLR allows for accurate pathological assessment of the tumor and surrounding liver tissue, enabling precise staging and identification of high-risk features. This information is crucial for determining appropriate adjuvant therapies and optimizing patient outcomes. Furthermore, LLR offers the benefit of improved perioperative and postoperative outcomes. Compared to locoregional ablative methods, LLR has been associated with shorter hospital stays, reduced postoperative pain, and faster recovery compared to open surgery. The minimally invasive nature of laparoscopic surgery minimizes surgical trauma, resulting in decreased morbidity and improved patient satisfaction. While locoregional ablative methods may be suitable for select patients with small, well-defined tumors, LLR provides a more comprehensive and curative approach for the management of left lateral segment hepatocellular carcinoma (HCC). However, further studies are needed to directly compare LLR with alternative treatment modalities and evaluate long-term outcomes, including overall survival and disease-free survival.

When HCC is located in a subcapsular lesion in the left lateral segment, there may be collateral thermal damage to adjacent organs (e.g., stomach, hepatic flexure of the colon, diaphragm, parietal peritoneum) during RFA compared with central lesions [20]. Peng et al. [11] reported that the efficacy and safety of percutaneous RF ablation were better than those of surgical resection in patients with HCC ≤2 cm in size, especially those with centrally located HCC. Therefore, the use of RFA for HCC located in the left lateral segment, which is a relatively subcapsular region compared with the central segment, should be considered carefully.

In the present study, we have shown that in patients with an AFP level of ≥20 ng/mL, the OS and DFS rates were greater in the LLR group than in the RFA group. The serum AFP level is an independent risk factor of HCC [4], but its prognostic role is limited in patients with well-compensated cirrhosis with a single small HCC who undergo treatment with curative intent [21]. An et al. [22] reported that the preoperative serum AFP level is an independent prognostic factor following surgical resection of HCC. In patients with HCC and a high AFP level, surgical resection can provide greater oncologic safety by achieving an appropriate resection margin, resulting in better OS and DFS rates compared with RFA [23]. There are several limitations to this study. First, the patient groups were limited to those with Child–Pugh class A and small HCCs (≤3 cm) in order to balance both groups, but the sample size was small, and propensity score matching was not performed, which may have resulted in confounding factors affecting the outcomes between the LLR and RFA groups. Second, the histopathologic diagnosis of HCC was not assessed in the RFA group. Since liver resection had better outcomes than RFA in patients with AFP levels ≥20 ng/mL, this difference in survival may be due to the inclusion of dysplastic nodules in the RFA group that were not histologically confirmed. The higher diagnostic probability of HCC in patients with AFP levels of ≥20 ng/mL may also contribute to the difference in survival.

## 5. Conclusions

LLR showed superior OS and DFS compared to RFA in patients with a single small HCC situated in the left lateral segment of the liver. LLR can be considered for patients with an α-fetoprotein level of ≥20 ng/mL. Further investigations, especially larger prospective clinical trials, are necessary to reach a definitive conclusion.

## Figures and Tables

**Table 1:** Baseline characteristics of the patients.

	LLR (*n* = 36)	RFA (*n* = 40)	*p*-Value
Sex (F/M)	11 (30.6%)/25 (69.4%)	6 (15%)/34 (85%)	0.112
Age, median (IQR)	57.8 (±11.70)	61.6 (±13.72)	0.170
Underlying liver disease			
HBV	30 (83.3%)	31 (77.5%)	
HCV	0 (0%)	1 (2.5%)	
Alcohol	14 (38.0%)	7 (17.5%)	
NBNC	6 (16.7%)	8 (20%)	
BMI (kg/m^2^)	23.09 (±4.8)	22.86 (±3.15)	0.804
MELD score	7.36 (±1.7)	8.55 (±2.05)	0.080
AFP (ng/mL)	199.6 (±415.6)	29.4 (±68.2)	0.020
Size (cm)	2 (±0.57)	1.5 (±0.51)	0.003
Liver function test			
Total bilirubin (mg/dL)	0.85 (±0.3)	0.80 (±0.48)	0.629
PT INR	1.06 (±0.7)	1.13 (±0.10)	0.003
Albumin (g/dL)	4.4 (±0.3)	3.9 (±0.44)	0.001
AST (IU/L)	31.8 (±14.3)	55.7 (±51.6)	0.007
ALT (IU/L)	31.3 (±12.9)	72.4 (±78.9)	0.002

Abbreviations: AFP, α-fetoprotein; ALT, alanine aminotransferase; AST, aspartate aminotransferase; BMI, body mass index; F, female; HBV, hepatitis B viral infection; HCV, hepatitis C viral infection; IQR, interquartile range; LLR, laparoscopic liver resection; M, male; MELD, Model for End-Stage Liver Disease; NBNC, non-B, non-C hepatocellular carcinoma; INR, international normalized ratio; RFA, radiofrequency ablation.

**Table 2:** Comparison of periprocedural outcomes and recurrence patterns.

	LLR (*n* = 36)	RFA (*n* = 40)	*p*-Value
Overall complications	2 (5.6%)	6 (15%)	0.009
Organ injury	1 (liver)	3 (liver, stomach, T-colon)	
Fluid collection	1 (2.8%)	0 (0%)	
Pulmonary complication		1	
Other		2 (unknown fever)	
Hospital stay (days)	4.9 (±2.76)	2.4 (±2.88)	<0.001
Early recurrence (<6 months)	0 (0%)	4 (10%)	0.044
Recurrence pattern			0.023
None	27 (75%)	17 (42.5%)	
Intrahepatic	8 (22.2%)	21 (52.5%)	
Extrahepatic	0 (0%)	1 (2.5%)	
Both intrahepatic and extrahepatic	1 (2.8%)	1 (2.5%)	

Abbreviations: LLR, laparoscopic liver resection; RFA, radiofrequency ablation; T-colon, transverse colon.

**Table 3:** Baseline characteristics of patients with AFP levels of ≥20 ng/mL.

	LLR (*n* = 16)	RFA (*n* = 10)	*p*-Value
Sex (F/M)	6 (37.5%)/10 (62.5%)	1 (10%)/9 (90%)	0.999
Age, median (IQR)	53.6 (±13.9)	58.1 (±8.81)	0.363
Underlying liver disease			
HBV	9 (90%)	8 (80%)	
HCV	1 (10%)	0 (0%)	
Alcohol	7 (43.8%)	2 (20%)	
NBNC	0 (0%)	2 (20%)	
BMI (kg/m^2^)	22.38 (±6.4)	23.36 (±3.8)	0.665
MELD score	7.49 (±1.0)	8.2 (±2.82)	0.360
AFP (ng/mL)	443.44 (±537.5)	106.32 (±106.5)	0.026
Size (cm)	2 (±0.63)	1.4 (±0.34)	0.017
Liver function test			
Total bilirubin (mg/dL)	0.83 (±0.3)	0.58 (±0.26)	0.022
PT INR	1.07 (±0.9)	1.08 (±0.7)	0.865
Albumin (g/dL)	4.3 (±0.4)	3.7 (±0.5)	0.006
AST (IU/L)	35.3 (±19.8)	86.1 (±68.2)	0.045
ALT (IU/L)	31.9 (13.1)	96.9 (±64.9)	0.011

Abbreviations: AFP, α-fetoprotein; ALT, alanine aminotransferase; AST, aspartate aminotransferase; BMI, body mass index; F, female; HBV, hepatitis B viral infection; HCV, hepatitis C viral infection; IQR, interquartile range; LLR, laparoscopic liver resection; M, male; MELD, Model for End-Stage Liver Disease; NBNC, non-B, non-C hepatocellular carcinoma; INR, international normalized ratio; RFA, radiofrequency ablation.

**Table 4:** Comparison of periprocedural outcomes and recurrence patterns of patients with AFP levels of ≥20 ng/mL.

	LLR (*n* = 16)	RFA (*n* = 10)	*p*-Value
Overall complications	2 (12.5%)	1 (10%)	0.854
Organ injury	1 (6.25%, liver)	1 (10%, stomach)	
Fluid collection	0 (0%)	0 (0%)	
Pulmonary complication	1 (6.25%)	0 (0%)	
Others	0 (0%)	0 (0%)	
Hospital stay (days)	5.0 (±2.83)	1.8 (±0.48)	<0.001
Early recurrence (<6 months)	0 (0%)	1 (10%)	0.343
Recurrence pattern			0.009
None	11 (68.8%)	2 (20%)	
Intrahepatic	5 (31.3%)	7 (70%)	
Extrahepatic	0 (0%)	1 (10%)	
Both intrahepatic and extrahepatic	0 (0%)	0 (0%)	

Abbreviations: LLR, laparoscopic liver resection; RFA, radiofrequency ablation.

## Data Availability

The data presented in this study are available in insert article.

## References

[1] Bray F., Ferlay J., Soerjomataram I., Siegel R.L., Torre L.A., Jemal A. (2018). Global cancer statistics 2018: GLOBOCAN estimates of incidence and mortality worldwide for 36 cancers in 185 countries. CA-Cancer J. Clin..

[2] Jinjuvadia R., Salami A., Lenhart A., Jinjuvadia K., Liangpunsakul S., Salgia R. (2017). Hepatocellular Carcinoma: A Decade of Hospitalizations and Financial Burden in the United States. Am. J. Med. Sci..

[3] European Association for the Study of the Liver (2018). EASL Clinical Practice Guidelines: Management of hepatocellular carcinoma. J. Hepatol..

[4] Bai D.S., Zhang C., Chen P., Jin S.J., Jiang G.Q. (2017). The prognostic correlation of AFP level at diagnosis with pathological grade, progression, and survival of patients with hepatocellular carcinoma. Sci. Rep.-UK.

[5] Vivarelli M., Bellusci R., Cucchetti A., Cavrini G., De Ruvo N., Aden A.A., La Barba G., Brillanti S., Cavallari A. (2002). Low recurrence rate of hepatocellular carcinoma after liver transplantation: Better patient selection or lower immunosuppression?. Transplantation.

[6] Marrero J.A., Kulik L.M., Sirlin C.B., Zhu A.X., Finn R.S., Abecassis M.M., Roberts L.R., Heimbach J.K. (2018). Diagnosis, Staging, and Management of Hepatocellular Carcinoma: 2018 Practice Guidance by the American Association for the Study of Liver Diseases. Hepatology.

[7] Korean Liver Cancer Association (KLCA) and National Cancer Center (NCC) Korea (2022). 2022 KLCA-NCC Korea practice guidelines for the management of hepatocellular carcinoma. Clin. Mol. Hepatol..

[8] Yoon Y.S., Han H.S., Cho J.Y., Ahn K.S. (2010). Total laparoscopic liver resection for hepatocellular carcinoma located in all segments of the liver. Surg. Endosc..

[9] Guro H., Cho J.Y., Han H.S., Yoon Y.S., Choi Y., Periyasamy M. (2016). Current status of laparoscopic liver resection for hepatocellular carcinoma. Clin. Mol. Hepatol..

[10] Hong S.K., Lee K.W., Lee S., Hong S.Y., Suh S., Han E.S., Choi Y., Yi N.J., Suh K.S. (2022). Impact of tumor size on hepatectomy outcomes in hepatocellular carcinoma: A nationwide propensity score matching analysis. Ann. Surg. Treat. Res..

[11] Peng Z.W., Lin X.J., Zhang Y.J., Liang H.H., Guo R.P., Shi M., Chen M.S. (2012). Radiofrequency ablation versus hepatic resection for the treatment of hepatocellular carcinomas 2 cm or smaller: A retrospective comparative study. Radiology.

[12] Xu G., Qi F.Z., Zhang J.H., Cheng G.F., Cai Y., Miao Y. (2012). Meta-analysis of surgical resection and radiofrequency ablation for early hepatocellular carcinoma. World J. Surg. Oncol..

[13] Ko S.E., Lee M.W., Ahn S., Rhim H., Kang T.W., Song K.D., Kim J.M., Choi G.S., Cha D.I., Min J.H. (2022). Laparoscopic Hepatic Resection Versus Laparoscopic Radiofrequency Ablation for Subcapsular Hepatocellular Carcinomas Smaller Than 3 cm: Analysis of Treatment Outcomes Using Propensity Score Matching. Korean J. Radiol..

[14] Lee J., Jin Y.J., Shin S.K., Kwon J.H., Kim S.G., Suh Y.J., Jeong Y., Yu J.H., Lee J.W., Kwon O.S. (2022). Surgery versus radiofrequency ablation in patients with Child- Pugh class-A/single small (</=3 cm) hepatocellular carcinoma. Clin. Mol. Hepatol..

[15] Song J., Wang Y., Ma K., Zheng S., Bie P., Xia F., Li X., Li J., Wang X., Chen J. (2016). Laparoscopic hepatectomy versus radiofrequency ablation for minimally invasive treatment of single, small hepatocellular carcinomas. Surg. Endosc..

[16] Kim J.M., Kang T.W., Kwon C.H., Joh J.W., Ko J.S., Park J.B., Rhim H., Lee J.H., Kim S.J., Paik S.W. (2014). Single hepatocellular carcinoma </= 3 cm in left lateral segment: Liver resection or radiofrequency ablation?. World J. Gastroenterol..

[17] Huang J., Yan L., Cheng Z., Wu H., Du L., Wang J., Xu Y., Zeng Y. (2010). A randomized trial comparing radiofrequency ablation and surgical resection for HCC conforming to the Milan criteria. Ann. Surg..

[18] Jin S., Tan S., Peng W., Jiang Y., Luo C. (2020). Radiofrequency ablation versus laparoscopic hepatectomy for treatment of hepatocellular carcinoma: A systematic review and meta-analysis. World J. Surg. Oncol..

[19] Vivarelli M., Guglielmi A., Ruzzenente A., Cucchetti A., Bellusci R., Cordiano C., Cavallari A. (2004). Surgical resection versus percutaneous radiofrequency ablation in the treatment of hepatocellular carcinoma on cirrhotic liver. Ann. Surg..

[20] Rhim H. (2005). Complications of radiofrequency ablation in hepatocellular carcinoma. Abdom. Imaging.

[21] Giannini E.G., Marenco S., Borgonovo G., Savarino V., Farinati F., Del Poggio P., Rapaccini G.L., Di Nolfo M.A., Benvegnu L., Zoli M. (2012). Alpha-fetoprotein has no prognostic role in small hepatocellular carcinoma identified during surveillance in compensated cirrhosis. Hepatology.

[22] An S.L., Xiao T., Wang L.M., Rong W.Q., Wu F., Feng L., Liu F.Q., Tian F., Wu J.X. (2015). Prognostic Significance of Preoperative Serum Alpha- fetoprotein in Hepatocellular Carcinoma and Correlation with Clinicopathological Factors: A Single-center Experience from China. Asian Pac. J. Cancer Prev..

[23] Kim S., Yoon C.J., Cho J.Y., Han H.S., Yoon Y.S., Lee H.W., Lee J.S., Kim M., Lee B., Ahn S. (2022). Comparative long-term outcomes of laparoscopic hepatectomy and radiofrequency ablation for hepatocellular carcinoma located in the anterolateral segments of the liver. J. Hepatobiliary Pancreat Sci..

